# Multifaceted Role of Social Media in Healthcare: Opportunities, Challenges, and the Need for Quality Control

**DOI:** 10.7759/cureus.39111

**Published:** 2023-05-16

**Authors:** Madhan Jeyaraman, Swaminathan Ramasubramanian, Shanmugapriya Kumar, Naveen Jeyaraman, Preethi Selvaraj, Arulkumar Nallakumarasamy, Suresh K Bondili, Sankalp Yadav

**Affiliations:** 1 Orthopaedics, ACS Medical College and Hospital, Dr MGR (M.G.Ramachandran) Educational and Research Institute, Chennai, IND; 2 Medicine, Government Medical College, Omandurar Government Estate, Chennai, IND; 3 Respiratory Medicine, Sri Lalithambigai Medical College and Hospital, Dr MGR (M.G.Ramachandran) Educational and Research Institute, Chennai, IND; 4 Orthopaedics, Shri Sathya Sai Medical College and Research Institute, Sri Balaji Vidyapeeth, Nellikuppam, IND; 5 Community Medicine, Sri Lalithambigai Medical College and Hospital, Dr MGR (M.G.Ramachandran) Educational and Research Institute, Chennai, IND; 6 Orthopaedics and Traumatology, All India Institute of Medical Sciences, Bhubaneswar, Bhubaneswar, IND; 7 Orthopedics, Institute of Medical Sciences, Banaras Hindu University, Varanasi, IND; 8 Medical Oncology, Kauvery Hospital, Chennai, IND; 9 Medicine, Shri Madan Lal Khurana Chest Clinic, Moti Nagar, New Delhi, IND

**Keywords:** medical education, patient-centred care, latest research, healthcare, effects of social media

## Abstract

Social media, leveraging Web 2.0 technologies, plays a vital role in healthcare, medical education, and research by fostering collaboration and enabling research dissemination. Healthcare professionals use these platforms to improve public health literacy, but concerns about misinformation and content accuracy persist. In 2023, platforms like Facebook (Meta Platforms, Inc., Menlo Park, California, United States), YouTube (Google LLC, Mountain View, California, United States), Instagram (Meta Platforms, Inc.), TikTok (ByteDance Ltd, Beijing, China), and Twitter (X Corp., Carson City, Nevada, United States) have become essential in healthcare, offering patient communication, professional development, and knowledge-sharing opportunities. However, challenges such as breaches of patient confidentiality and unprofessional conduct remain. Social media has transformed medical education, providing unique networking and professional development opportunities. Further studies are needed to determine its educational value. Healthcare professionals must follow ethical and professional guidelines, particularly regarding patient privacy, confidentiality, disclosure rules, and copyright laws. Social media significantly impacts patient education and healthcare research. Platforms like WhatsApp (Meta Platforms, Inc.) effectively improve patient compliance and outcomes. Yet, the rapid dissemination of false news and misinformation on social media platforms presents risks. Researchers must consider potential biases and content quality when extracting data. Quality control and regulation are crucial in addressing potential dangers and misinformation in social media and healthcare. Stricter regulations and monitoring are needed due to cases of deaths resulting from social media trends and false news spread. Ethical frameworks, informed consent practices, risk assessments, and appropriate data management strategies are essential for responsible research using social media technologies. Healthcare professionals and researchers must judiciously use social media, considering its risks to maximize benefits and mitigate potential drawbacks. By striking the right balance, healthcare professionals can enhance patient outcomes, medical education, research, and the overall healthcare experience.

## Introduction and background

Social media includes an array of digital communication platforms, including but not limited to Facebook (Meta Platforms, Inc., Menlo Park, California, United States), WhatsApp (Meta Platforms, Inc.), Instagram (Meta Platforms, Inc.), Twitter (X Corp., Carson City, Nevada, United States), YouTube (Google LLC, Mountain View, California, United States), Instagram (Meta Platforms, Inc.), TikTok (ByteDance Ltd, Beijing, China), LinkedIn (LinkedIn Corporation, Sunnyvale, California, United States), Quora (Quora, Inc., Mountain View, California, United States), Discord (Discord Inc., San Francisco, California, United States), etc. which facilitate the creation and sharing of information and ideas on levels ranging from a peer-to-peer basis to a broader scale. These platforms are built upon the principles of Web 2.0 technologies and have emerged as essential tools in healthcare, medical education, and research [1]. The utilization of social media in clinical education offers valuable insights into curricula, which enables students to be better equipped before commencing their journey into medical education [2-4]. These platforms prove beneficial in acquiring information throughout one's academic pursuits and beyond [2-4]. These platforms have significantly impacted academic research by providing opportunities for collaboration, providing access to extensive data sources, and facilitating recruitment. Furthermore, social media serves as a medium for the dissemination of research findings to a wider audience [2-4]. Healthcare professionals are also using social media to educate the general public on various healthcare topics, thereby improving health literacy [2-4]. However, concerns have been raised regarding the dissemination of false information and the dearth of regulatory mechanisms to ensure the accuracy of the content shared on these platforms. This review article seeks to elucidate the importance of social media in healthcare, medical education, and research, while concurrently addressing the potential drawbacks associated with its usage.

## Review

Social media platforms

**Figure 1:**
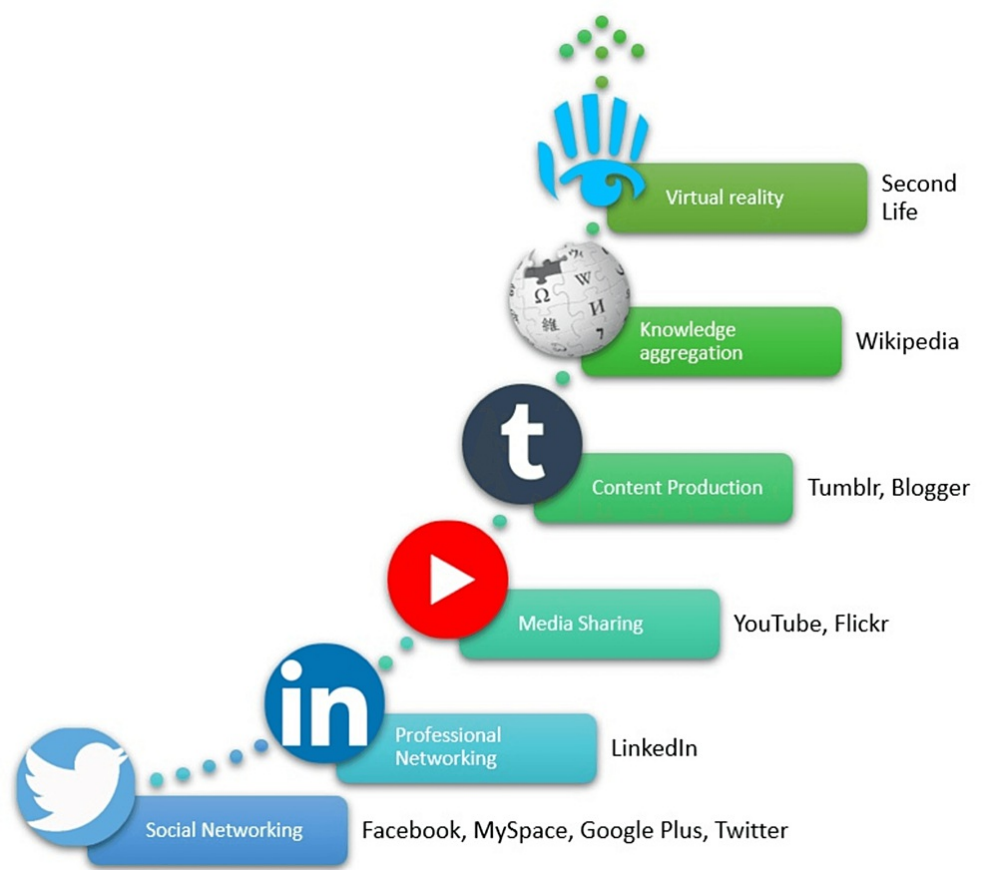
Summary of social media platforms Image credit: Madhan Jeyaraman

Over the years, social media platforms have experienced substantial growth and diverse levels of user engagement. In 2023, the predominant platforms utilized by consumers are Facebook (69%), YouTube (57%), Instagram (45%), TikTok (33%), and Twitter (30%) [5]. The global user base of social media has exhibited an upward trajectory, growing from 4.2 billion users in January 2021 to 4.62 billion users by January 2022, marking a 10.1% year-over-year increase [6]. A modest growth of 3%, equivalent to an addition of 137 million users, has been observed as of January 2023. The social media and networking platforms are concisely represented in Figure 1.

Facebook, the largest platform with 2.9 billion monthly active users, is being used in healthcare for purposes such as facilitating patient support groups, disseminating information, and promoting awareness campaigns. YouTube, distinguished by its video-centric content, serves as an invaluable resource for patient education, expert interviews, and the communication of health-related updates. Instagram, predominantly preferred by younger audiences, is conducive to visual storytelling, promoting wellness and healthy lifestyles, and establishing connections with healthcare influencers. Twitter, characterized by its real-time updates, is an outstanding platform for healthcare organizations to share news, interact with patients, and engage in health-related discussions.

Social media platforms have emerged as essential tools for healthcare marketers and professionals to connect with patients and disseminate health-related information [7]. As of the fourth quarter of 2022, Facebook is the largest online social network globally, boasting approximately 2.9 billion monthly active users, making it the most widely used platform by marketers worldwide. Instagram ranks second, with 79% of the users using it for e-commerce. Twitter has approximately 556 million monthly active users worldwide, with 53% of users employing the platform to access the latest news [8].

Social media and its importance in healthcare

Social media platforms have emerged as indispensable tools in the healthcare sector, playing a crucial role in disseminating health-related information, medical research, education, patient communication, and professional development. With a significant percentage of patients utilizing social media to search for and share health information, these platforms have gained prominence in various health topics, such as vaccines, drugs, smoking, noncommunicable diseases, pandemics, eating disorders, and medical treatments [9-11]. Medical research has benefitted from the numerous health-related applications offered by social media, including health interventions, health campaigns, medical education, and disease outbreak surveillance [12]. Furthermore, the relationship between social media use and mental health has garnered significant interest, presenting potential as an intervention platform for individuals with mental disorders [13-15].

In medical education, social media usage has expanded among healthcare professionals and organizations, with younger populations' media device use indicating that social media can play a critical role in delivering medical education to future healthcare professionals [16]. Social media enables users to create and share content, engage in social networking, and participate in active learning, fostering self-reflection and knowledge creation [17,18]. Despite its potential benefits, determining the educational value of social media in medical education requires further empirical evaluative studies. Medical education curricula currently lack comprehensive guidance on the meaningful use and deployment of social media. Clinicians and institutions must evolve to embrace social media platforms for medical education while adhering to the same ethical standards as in-person patient interactions [19,20].

Ramasubramanian et al. analyzed the accuracy of Instagram posts, finding that 88.1% of the posts on stroke were factually accurate, although healthcare professionals' contributions to social media posts were minimal [21]. von Muhlen and Ohno-Machado's systematic review discovered an increased use of social media among students and healthcare professionals, with Facebook emerging as the most commonly used platform [22]. They also highlighted potential pitfalls, such as breaches of patient confidentiality and unprofessional conduct. Marshal et al. emphasized the need for personal and professional content separation online and the establishment of guidelines to address this issue [23]. Lambert et al. underscored the necessity for healthcare organizations to have proactive policies in place to address social media use by employees, both at work and on personal accounts [24]. The complexity of social media and the need for guidelines to be modified as technology advances stress the importance of healthcare professionals using social media responsibly and maintaining the public's trust [25].

Social media in medical education

Social media has become an indispensable resource for medical education, offering unique opportunities for professional development, networking, and knowledge sharing. In nursing education, Peck emphasized the potential of social media as a teaching tool while underscoring the importance of protecting student privacy and providing appropriate guidelines for its use to ensure safety in the learning environment [26]. She advocated for educational institutions to establish standards of conduct and privacy considerations and for faculty to educate students on the same.

Rukavina et al. identified three key benefits of social media on the e-professionalism of healthcare professionals, namely professional networking and collaboration, professional education and training, and patient education and health promotion [27]. They also recognized five dangers, including loosening accountability, compromising confidentiality, blurred professional boundaries, depiction of unprofessional behavior, and legal issues and disciplinary consequences. The review acknowledged that educational curricula regarding e-professionalism and barriers that affect the use of social media by healthcare professionals could benefit from improvements and changes.

Bernhardt et al. suggested that social media platforms present immense potential for professional development and networking, despite the potential risks, and should be used judiciously to maximize the benefits [28]. They contend that the advantages of social media use for professional advancement outweigh the risks, urging professionals to leverage these platforms. Betts et al. revealed that nurse practitioners and physician assistants use online professional communities at a similar rate to primary care physicians and specialists, with almost half of the participants reporting that they utilize these communities to communicate with colleagues regarding patient care [29].

Bryan et al. found that parents of pediatric patients perceived blog posts written in the third-person objective voice as more reliable and accurate than those written in the personal or mixed voice [30]. The topic of the post also affected parental ratings, as sleep-related posts were deemed more accurate than vaccine-related posts, leading the authors to recommend that pediatrician bloggers take into account both the narrative voice and the topic they are addressing to strengthen parental trust in the veracity of their content [30]. The overview of studies on social media in medical education is tabulated in Table 1.

**Table 1:** Overview of social media in medical education

Author(s)	Study Focus	Benefits of Social Media in Medical Education	Limitations and Risks
Peck [26]	Social media in nursing education	Teaching tool, student privacy protection, appropriate guidelines for use	Need for standards of conduct and privacy considerations, potential safety concerns in learning environment
Rukavina et al. [27]	E-professionalism of healthcare professionals	Professional networking and collaboration, professional education and training, patient education, and health promotion	Loosening accountability, compromising confidentiality, blurred professional boundaries, depiction of unprofessional behavior, legal issues, and disciplinary consequences
Bernhardt et al. [28]	Professional development and networking	Immense potential for professional development and networking, advantages outweigh risks	Potential risks, need for judicious use to maximize benefits
Betts et al. [29]	Use of online professional communities	Similar usage rates among various healthcare professionals, communication with colleagues about patient care	Not specified
Bryan et al. [30]	Parental perception of blog posts	Improved reliability and accuracy with third-person objective voice, sleep-related posts deemed more accurate	Personal or mixed voice perceived as less reliable, vaccine-related posts perceived as less accurate

Social media platforms in healthcare

In a study by Ramasubramanian et al., Instagram's role in healthcare communication was analyzed, finding that 88.1% of the posts on stroke were factually accurate, yet the contribution of healthcare professionals in social media posts remained minimal [21]. von Muhlen and Ohno-Muchado found Facebook to be the most commonly used platform among healthcare professionals and students, although they also highlighted potential pitfalls such as breaches of patient confidentiality and unprofessional conduct [22]. Heras-Pedrosa et al. further emphasized the significance of Twitter and Facebook as essential tools for information dissemination, relationship establishment, and user engagement in healthcare contexts [31]. Lambert et al. stressed the need for proactive policies to address social media use by employees in healthcare organizations [24].

The complexity of social media requires ongoing modification of guidelines as technology advances [25], with healthcare professionals urged to use social media responsibly to maintain public trust. Ethical and professional guidelines should be followed when using social media, such as patient/client privacy and confidentiality, disclosure rules, and copyright laws [32]. Web 1.0, often referred to as the "static web," signifies the early stage of the internet characterized by predominantly static, read-only content and limited user interaction. Web 2.0, conversely, represents the evolution of the internet towards dynamic, user-generated content, fostering greater interactivity and collaboration among users through social networking, blogs, and multimedia-sharing platforms [33]. Hardey noted that Web 2.0 offers opportunities for disseminating health information, creating new data sources, and generating new questions and dilemmas [34].

Patient education and social media

Social media plays a crucial role in patient education, addressing patient needs, and promoting patient compliance. Mouelhi et al. highlight the need for accessible and easy-to-find medical information on social media to keep patients informed [35], while Marshal et al. described the need for personal and professional content separation online and the development of guidelines to address this issue [23]. Elmously et al. stress the importance of using social media to provide accessible healthcare information for all stakeholders and to unify patients with similar diseases [36].

Bryan et al. found that the narrative voice and topic of blog posts affected parental trust in their content, with sleep-related posts deemed more accurate than vaccine-related posts [30]. They recommended that pediatrician bloggers take into account both the narrative voice and the topic they are addressing to strengthen parental trust in the veracity of their content. Zotti et al. demonstrated that engaging adolescent patients through WhatsApp activity could improve orthodontic treatment compliance and outcomes, increasing regularity in wearing removable retainers, attendance to follow-up schedules, and long-term orthodontic stability [37].

Social media in healthcare research

Ramasubramanian et al. and Alves et al. demonstrate the use of social media data in healthcare research and its potential as a stimulant for research questions [21,38]. Abroms called for the modification of social media structures to be health-enhancing, similar to how we have crafted our built environment, as social media sites represent the primary social institutions shaping modern life, thereby requiring the provision of health-promoting policies, programs, and information to optimize public health [39]. D’Souza et al. proposed steps to approach studies using data from social media, highlighting the importance of framing research questions, identifying the social media outlet and selecting content, extracting data systematically, assessing content quality and sources of bias, analyzing data, and interpreting study findings [19]. Zhou et al. presented a conceptual framework for managing health information through social media and identified research challenges and unexplored topics in the field, asserting that numerous inquiries regarding data analytics, ethics, governance, privacy, confidentiality, professionalism, and information quality remain unresolved [40].

Sohn et al. introduced the Crowdsourced Health Experience-ontologies-based healthcare Knowledge Creation (CHEKC) framework, which leverages experience-ontologies to integrate healthcare knowledge and provides patients with healthcare information based on similar healthcare experiences, symptoms, and conditions, demonstrating superior efficiency and accuracy over the PatienstLikeMe.com (PLM) framework through two experiments, underscoring the potential of social media in research and data collection [41]. Numerous studies have highlighted the importance of using social media responsibly in healthcare research, emphasizing the need for ethical and professional guidelines, as well as proactive policies by healthcare organizations [24,32]. In addition to these concerns, social media has also been shown to disseminate both accurate and inaccurate information, such as during the coronavirus disease 2019 (COVID-19) pandemic, causing fear or panic among the public [42].

Despite potential drawbacks, various papers have demonstrated the benefits of using social media in healthcare research. For example, Davies et al. found that internet-delivered interventions effectively increased physical activity [43], while Zotti et al. suggested that engaging adolescent patients through WhatsApp improved orthodontic compliance and outcomes [37]. However, given the rapid dissemination of false news on platforms such as Twitter [44], healthcare professionals need to use social media judiciously and be aware of the risks associated with its use. Researchers should be mindful of potential bias and the quality of content while extracting data from social media platforms, ensuring that the benefits of social media in healthcare research are maximized while mitigating potential risks [19].

Quality control and regulation of healthcare information in social media

Quality control and regulation of healthcare information in social media are crucial to address potential dangers and misinformation. Gabarron et al. discovered that social media had a significant impact on disseminating both accurate and inaccurate information about COVID-19, with misinformation ranging from 0.2% to 28.8% of posts, and all the studies that were included in their review evaluated the effects of such misinformation and reported that it caused fear or panic [42]. Farsi et al. concluded that while social media can be a useful tool for patients to improve their health and knowledge, it is important to be cautious and assess the credibility of the information obtained and its source [45]. There have been cases of deaths among the public resulting from social media trends, such as the Tide Pod Challenge [46], Benadryl Challenge [47], or codeine intoxication following an internet recipe [48], which call for stricter regulation on social media. Vosoughi et al. revealed that false news stories spread significantly faster, further, and more extensively than true news on Twitter between 2006 and 2017, with false political news exhibiting the most significant effects [44]. This finding underscores the need for stricter regulations and monitoring of social media. 

Nebeker et al. examined the ethical considerations associated with the use of mobile imaging, pervasive sensing, social media, and location tracking (MISST) technologies in behavioral research, highlighting the need for developing an ethical framework, informed consent practices, assessing potential risks to participants, and identifying appropriate data management strategies to guide the responsible design and ethical review of research using these technologies [49]. Tagliabue et al. emphasize the crucial role of mass media in providing clear, accessible, and evidence-based public health information during the COVID-19 pandemic while highlighting the need for better coordination between the medical community, governments, and mass media to limit the spread of fake news and encourage compliance with accurate guidelines [50]. Pascali et al. discussed the dangers of social media in the sense that it may lead to tampering with drugs to increase their potency, which is usually not tested and dangerous and may cause injuries or death, indicating the need for stricter regulations to prevent abuse [51]. Patel and Jermacane explored the utilization of social media in healthcare, including travel medicine, as a means of providing timely information and facilitating collaboration while highlighting potential drawbacks, such as dissemination of low-quality information and privacy breaches, underscoring the importance for healthcare practitioners to exercise caution while leveraging social media in their practice [52].

Striking the right balance in social media

**Figure 2:**
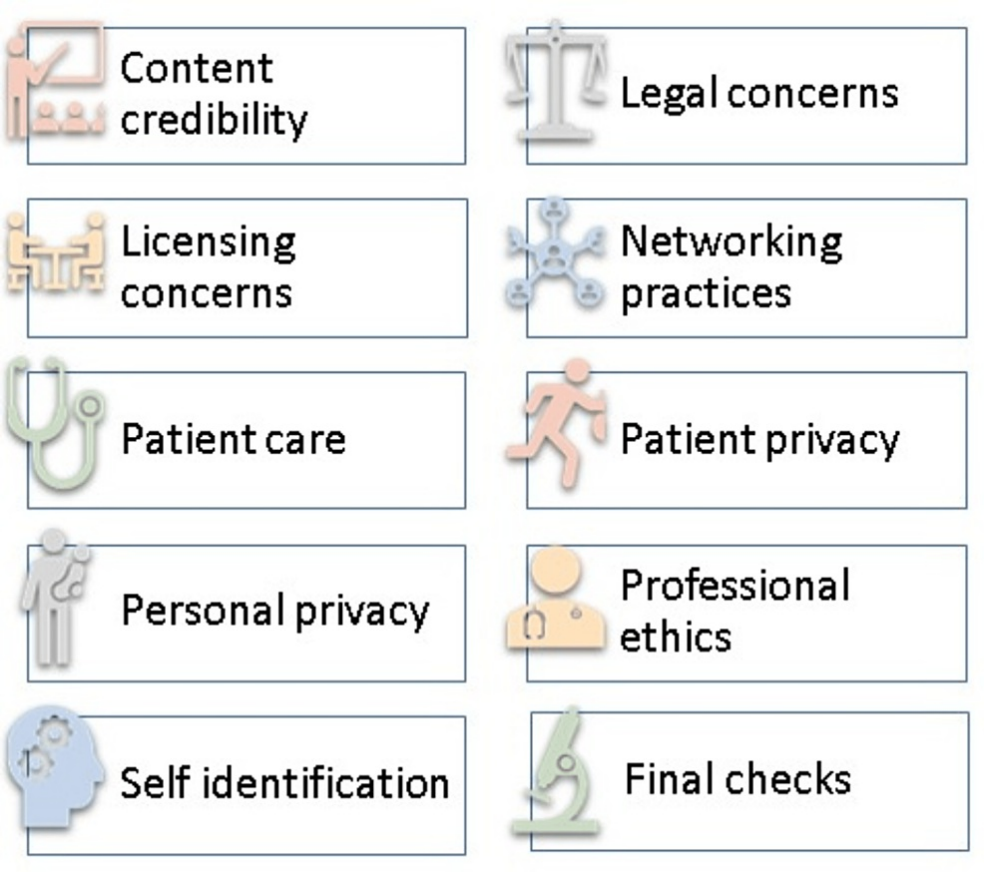
Tips for using social media in health education and research Image Credit: Madhan Jeyaraman

Social media has become an indispensable tool in healthcare, providing valuable opportunities for patient education, medical education, healthcare research, and professional development. However, it is crucial to strike the right balance between maximizing the benefits and mitigating the potential risks associated with its use. For patients, it is essential to be cautious and assess the credibility of the information obtained and its source [45]. Healthcare professionals must maintain the public's trust by using social media responsibly [25] and adhering to ethical and professional guidelines, such as patient/client privacy and confidentiality, disclosure rules, and copyright laws [32]. They should be mindful of their social media presence and use the available privacy settings to maintain their professionalism [53]. Healthcare organizations must have proactive policies in place to address social media use by employees, both at work and on personal accounts [24]. They should also provide training and support for healthcare professionals on the responsible and effective use of social media. Research using social media data should involve framing a research question, identifying the social media outlet and selecting content, extracting data systematically, assessing the quality of content and sources of bias, analyzing the data, and interpreting the study findings [40]. Ethical considerations must be taken into account, such as developing an ethical framework, informed consent practices, assessing potential risks to participants, and identifying appropriate data management strategies [49]. Table 2 delineates potential institutional policies that may be enforced to ensure the appropriate use of social media, while Figure 2 offers a selection of tips for effectively utilizing social media platforms in the realms of healthcare and research. In the face of the COVID-19 pandemic, the crucial role of mass media in providing clear, accessible, and evidence-based public health information was emphasized, highlighting the need for better coordination between the medical community, governments, and mass media to limit the spread of fake news and encourage compliance with accurate guidelines [50].

**Table 2:** Health care institutions' policies on the usage of social media

Policies to be followed
Need to address harassment, incorrect termination, damage to reputation of association or breach in confidential information.
Employee's access to networking arena ought to be limited or monitored.
Define employees’ responsibilities when witnessing inappropriate use of social media.
Need of an hour calls for drafting of policies regularizing use of organizational email or any logos.
Inappropriate use of social media calls for defining of disciplinary actions.
Access to social media during working premises and purpose of usage needs appropriate authorization.
Medical staff should disclose conflicts of interest in case if any.
Medical staff and employees should familiarize themselves with state guidelines guarding privacy of patient.
Medical staff should portray a disclaimer in case if they are not presenting on behalf of their organization.
All the employees and students should be oriented towards adherence to institutional policy on using social media sources.
Policies regarding consent and sharing of sensitive patient's information on social media calls for expansion.

## Conclusions

Social media holds immense potential for healthcare, but it is essential to strike the right balance by using it responsibly, adhering to ethical and professional guidelines, and implementing effective policies and regulations. By doing so, healthcare professionals, organizations, and patients can harness the power of social media to improve patient outcomes, advance medical knowledge, and enhance the overall healthcare experience. The present article highlights the importance of social media platforms, which play a crucial role in healthcare, medical education, and research by enabling collaboration, communication, knowledge sharing, patient communication, professional development, and networking. However, they are also associated with challenges like breaches of patient confidentiality, unprofessional conduct, and misinformation, making ethical and professional guidelines essential. Also, social media significantly impacts patient education and healthcare research but is prone to the spread of false news and misinformation. Therefore, quality control, stricter regulations, and ethical frameworks are necessary to address potential dangers and misinformation in social media and healthcare. Healthcare professionals must judiciously use social media to maximize benefits and minimize risks, thus enhancing patient outcomes, medical education, research, and the overall healthcare experience.
